# Structural Modification and Biological Activity of Polysaccharides

**DOI:** 10.3390/molecules28145416

**Published:** 2023-07-14

**Authors:** Ting Zhao, Min Yang, Lina Ma, Xinglong Liu, Qiteng Ding, Guodong Chai, Yang Lu, Hewei Wei, Shuai Zhang, Chuanbo Ding

**Affiliations:** 1 College of Traditional Chinese Medicine, Jilin Agriculture Science and Technology College, Jilin 132101, China; lyguiwandingding@163.com (T.Z.); yangmin@ilnku.edu.cn (M.Y.); malina@ilnku.edu.cn (L.M.); xinglongliu1221@126.com (X.L.); 2 Scientific and Technological Innovation Center of Health Products and Medical Materials with Characteristic Resources of Jilin Province, Jilin Agricultural University, Changchun 130118, China; ding152778@163.com (Q.D.); cgdong1008611@163.com (G.C.); luyang01023@163.com (Y.L.); www03211@126.com (H.W.); 3 College of Chinese Medicinal Materials, Jilin Agricultural University, Changchun 130118, China; 4 College of Resources and Environment, Jilin Agricultural University, Changchun 130118, China

**Keywords:** polysaccharide, structural modification, structural characterization, biological activity

## Abstract

Natural polysaccharides are macromolecular substances with a wide range of biological activities. The structural modification of polysaccharides by chemical means can enhance their biological activity. This paper reviews the latest research reports on the chemical modification of natural polysaccharides. At present, the modification methods of polysaccharides mainly include sulfation, phosphorylation, carboxymethylation, socialization, methylation and acetylation. The chemical and physical structures of the modified polysaccharides were detected via ultraviolet spectroscopy, FT-IR, high-performance liquid chromatography, ultraviolet spectroscopy, gas chromatography–mass spectrometry, nuclear magnetic resonance and scanning electron microscopy. Modern pharmacological studies have shown that the modified polysaccharide has various biological activities, such as antioxidant, antitumor, immune regulation, antiviral, antibacterial and anticoagulant functions in vitro. This review provides fresh ideas for the research and application of polysaccharide structure modification.

## 1. Introduction

Polysaccharides are macromolecular polymers widely found in plants, animals and microorganisms [1]. Polysaccharides generally consist of more than 10 monosaccharides linked by glycosidic bonds in linear or branched chains [2]. Polysaccharides composed of the same monosaccharides are called homopolysaccharides, such as starch [3]. Polysaccharides composed of different monosaccharides are called heteropolysaccharides, such as gum arabic, which is composed of pentose and galactose. Polysaccharides are generally insoluble in water, have no sweet taste, cannot form crystals, and have no reduction or mutarotation. At present, polysaccharide extraction methods include hot water extraction, acid extraction, alkali extraction and enzyme extraction [4]. A large number of studies have shown that polysaccsharides have anti-oxidation [5], anti-tumor [6], anti-coagulation [7], anti-cancer [8], hypoglycemic [9] and immunomodulatory activities [10]. This wide range of biological activities suggests that polysaccharides have potentially great advantages in the fields of food and medicine.

Various natural polysaccharides exhibit only weak biological activity. Therefore, in order to expand the biological activity of natural polysaccharides, the method of chemical modification is used to change their chemical structure and conformation [11,12]. At present, the modification methods of polysaccharides mainly include sulfation, phosphorylation, carboxymethylation, socialization, methylation and acetylation. Changing polysaccharides’ structure can not only improve their biological characteristics but sometimes also confer new biological activities [13]. Different modification methods produce different degrees of substitution (DS) and have different effects on activity [14].

Cyclodextrins are naturally occurring, non-toxic and biodegradable cyclic oligosaccharides [15,16,17]. Although many cyclodextrin derivatives are commercially available, their prices are in the range of those of fine chemicals and, therefore, they are still often synthesized in laboratories [18,19,20]. Modifying already substituted cyclodextrins with appropriate functional groups is much easier than optimizing the substitution for each desired new cyclodextrin derivative [21]. Therefore, artificial polysaccharides such as cyclodextrin also need to undergo structural modification to increase their biological activity.

**Figure 1:**
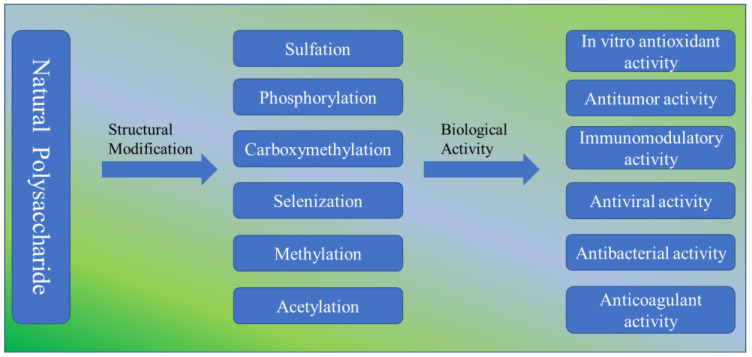
Structural modification and biological activity of natural polysaccharides.

The structural modification of natural polysaccharides provides additional ideas and possibilities for the application of polysaccharides. We reviewed the latest research progress on the chemical modification of polysaccharides, including their modification methods, structural characterization and biological activities (Figure 1).

## 2. Modification Method of Polysaccharide

### 2.1. Sulfation

**Figure 2:**
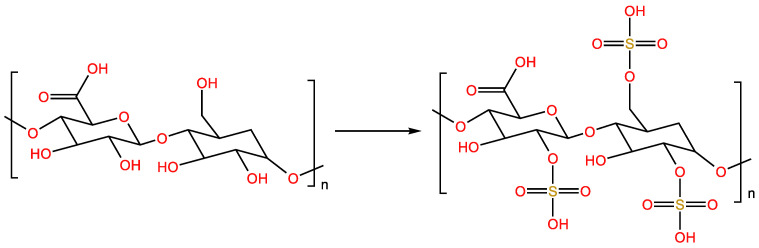
Sulfation modified polysaccharide reaction formula.

Polysaccharide sulfation is the chemical sulfonation of polysaccharide chains to form polyanions [22]. The sulfate is usually introduced at the hydroxyl groups at the C-1, 2, 3, 4 and 6 positions of the polysaccharide. The structure of sulfated polysaccharides is changed from that of the precursor polysaccharides, which may lead to changes in biological activity [23]. Commonly used polysaccharide sulfation methods include the clarifying acid–pyridine method, concentrated sulfuric acid method and sulfur trioxide–pyridine method (Figure 2).

#### 2.1.1. Chlorosulfonic Acid–Pyridine Method

The chlorosulfonic acid–pyridine method is the most commonly used sulfation method [24]. The sulfonated reagent is prepared in a low-temperature environment by adding a specific proportion of chlorosulfonic acid dropwise into anhydrous pyridine and stirring it continuously. The polysaccharide powder is stirred and dissolved in N,N-dimethylformamide, and then the surrounding reagent is added. After the reaction, the pH is adjusted to 7 with NaOH. Precipitation with alcohol, dialysis and lyophilization is performed to obtain the sulfated polysaccharides [23].

The DS of sulfated polysaccharides is affected by the ratio of the reagents, reaction time and temperature [10]. The reaction temperature has a greater influence, followed by the reaction time and the ratio of the sulfonated reagents [11]. The DS can be increased via the microwave-assisted synthesis of sulfated polysaccharides [25]. 4-Dimethylaminopyridine/dimethyl cyclohexyl carbodiimide, as a catalyst for the sulfonation of Artemisia polysaccharides, can improve the increase in the DS. In the process of sulfonation, polysaccharides are easily decomposed under high-temperature and acidic conditions, resulting in a decrease in the yield of sulfation. The yield and DS of sulfated polysaccharides prepared using the chlorosulfonic acid–pyridine method are high, this being a commonly used preparation method.

#### 2.1.2. Concentrated Sulfuric Acid Method

The preparation process of the concentrated sulfuric acid method is similar to the preparation of the chlorosulfonic acid–pyridine method [26]. Concentrated sulfuric acid and pyridine were mixed in proportion to prepare an esterification reagent and stirred with n-butanol in an ice water bath. Then, ammonium sulfate was added to the reaction system, and polysaccharide powder was slowly added after mixing. The reactant was reacted at room temperature for 3 h, and the pH was adjusted to 7 with ice NaOH. Ethanol was added to precipitate the sulfated polysaccharides, and the precipitate was collected via centrifugation, dialyzed and freeze-dried to obtain the sulfated polysaccharides [10,11]. *Cordyceps gunnii* mycelium polysaccharides were used to prepare sulfated polysaccharides using a concentrated sulfuric acid method, and after infrared analysis, high-performance liquid chromatography and polyacrylamide gel electrophoresis analysis, it was proved that the sulfated polysaccharides were successful [27].

Compared with the chlorosulfonic acid–pyridine method, the concentrated sulfuric acid method has the characteristics of safe reagents. However, the disadvantage of this method is that the polysaccharides are easily degraded and carbonized by the concentrated sulfuric acid.

#### 2.1.3. Sulfur Trioxide–Pyridine Method

The sulfur trioxide–pyridine method is an effective method for the sulfation of polysaccharides, and the sulfate group is provided by SO_3_. It involves incorporating SO_3_ into pyridine to prepare an esterification reagent, dissolving polysaccharide powder in N,N-dimethylformamide, and adding the polysaccharide N,N-dimethylformamide solution to the esterification reagent. After stirring for 3 h, the pH is adjusted with NaOH and precipitated with ethanol to obtain the sulfated polysaccharides [28]. The preparation of the sulfated polysaccharides from Lycium barbarum polysaccharides is performed using a sulfur trioxide–pyridine esterification reagent in N,N-dimethylformamide. The reagents of the sulfur trioxide–pyridine method are dangerous, and the storage of SO_3_ limits the scope of application of the method to a certain extent.

### 2.2. Phosphorylation

**Figure 3:**
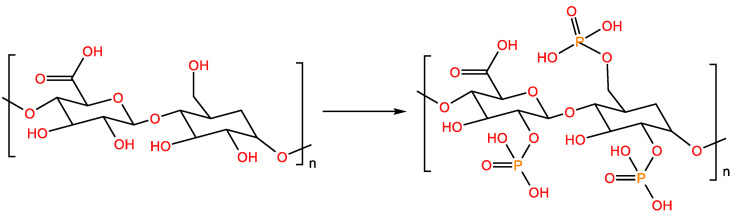
Phosphorylation-modified polysaccharide reaction formula.

Phosphorylated polysaccharides are widely distributed in nature and have complex structures. The types of natural phosphorylated polysaccharides are limited; hence, polysaccharides are often structurally modified to prepare fresh phosphorylated polysaccharides [29]. In the phosphorylation reaction, the hydroxyl groups of polysaccharides are replaced with phosphate groups. Phosphorylation introduces a dotted phosphate group, which changes the molecular weight of the polysaccharide and the conformation of the sugar chain, further increasing its water solubility [30]. Anhydrides, phosphorus oxychloride, phosphate and phosphorus pentoxide are usually used for the phosphorylation of polysaccharides (Figure 3).

#### 2.2.1. Anhydride Method

The polysaccharide powder was dissolved in dimethyl sulfoxide containing urea, and phosphoric acid was added dropwise to the solution and then immediately heated [31] to 100 °C and stirred for 6 h. After the temperature was cooled to room temperature, the mixture was dialyzed with distilled water to obtain phosphorylated polysaccharides [32]. During preparation, the dimethyl sulfoxide can be replaced with N,N-dimethylformamide, which will affect the synthesis rate. This method has low toxicity and a safe preparation process.

#### 2.2.2. Phosphorus Trioxide Method

Phosphorus trioxide is a highly reactive phosphorus oxychloride used for phosphorylated polysaccharides with a high DS. Phosphorus trioxide was added to anhydrous pyridine in a specific ratio in an ice water bath, and the phosphorylation reagent was prepared via constant stirring. The polysaccharide powder was added to anhydrous formamide at room temperature, and the anhydrous pyridine was added dropwise and stirred for 30 min until the polysaccharide was completely dissolved. The phosphorylation reagent was added dropwise to the polysaccharide formamide solution, and after 3 h of reaction, the mixture was cooled and neutralized with NaOH. The phosphorylated polysaccharides were precipitated with ethanol, dialyzed and freeze-dried [33].

#### 2.2.3. Phosphate Method

The preparation of phosphorylated polysaccharides can be achieved using the phosphate method with sodium tripolyphosphate and sodium trimetaphosphate [34]. One dissolves specific proportions of sodium tripolyphosphate and sodium trimetaphosphate in water, adding polysaccharide powder and allowing it to dissolve. The pH of the solution is adjusted to 9 with NaHCO_3_ and reacted at 80 °C for 5 h. The phosphorylated polysaccharides are precipitated with ethanol and dialyzed to lyophilize. Preparing phosphorylated polysaccharide using this method is safer.

#### 2.2.4. Phosphorus Pentoxide Method

Phosphorus pentoxide is a commonly used phosphorylation reagent with acceptable safety. The polysaccharide phosphorylation reaction uses methanesulfonic acid as a solvent and needs to be reacted in a low-temperature environment. However, the low solubility of phosphorus pentoxide and the easy decomposition of polysaccharides in a strong acid environment limit the application of this method [35].

### 2.3. Carboxymethylation

**Figure 4:**
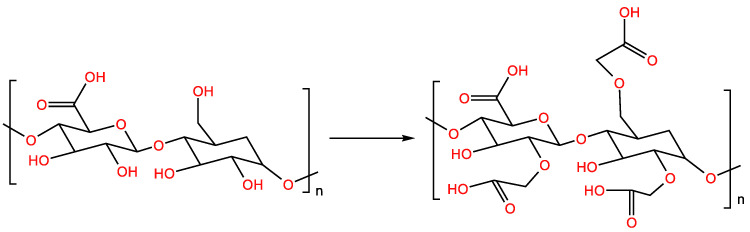
Carboxymethylation-modified polysaccharide reaction formula.

The carboxymethylation of polysaccharides serves to replace the hydroxyl groups of polysaccharides with carboxymethyl groups in order to change the conformation of polysaccharides (Figure 4), thereby enhancing the water solubility of the polysaccharides and further improving their biological activity [36]. The carboxymethylation reagent was prepared by adding chloroacetic acid to 20% NaOH and isopropanol. The polysaccharide powder was suspended in isopropanol and stirred for 30 min, and then 20% NaOH was added and stirred at room temperature for 3 h. Finally, carboxymethylation reagent was added while stirring and left to react at 60 °C for 4 h, and carboxymethyl polysaccharide was obtained via ethanol precipitation [37]. The DS is most affected by chloroacetic acid, and the amount of chloroacetic acid added is considered during preparation to prepare carboxymethyl polysaccharides [38].

### 2.4. Selenization

**Figure 5:**
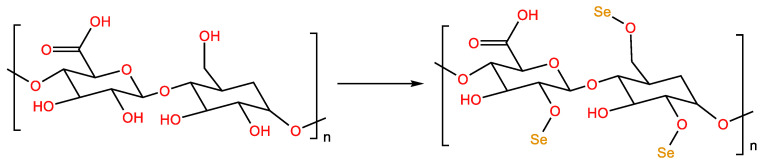
Selenization-modified polysaccharide reaction formula.

Selenium is an essential trace element for the growth of various organisms and has biological activities such as anti-oxidation, hypoglycemic, and hypolipidemic functions [39]. However, the toxicity of inorganic selenium is much higher than that of organic selenium. Natural selenium-containing polysaccharides exist in plants or microorganisms in low concentrations. Selenium polysaccharide is a new type of functional polysaccharide, which combines inorganic selenium and organic selenium formed by polysaccharides [39]. The selenization methods of polysaccharides mainly include the use of sodium nitrate selenite [40], nitric acid–selenous acid [41], glacial acetic acid–selenous acid, glacial sodium acetate–sodium selenite and selenium oxychloride [42]. The sodium nitrate selenite method is commonly used in the selenization of polysaccharides because of its simple reaction conditions and high selenization efficiency. One adds polysaccharide powder to HNO_3_, followed by stirring at room temperature for 0.5 h, and then adds Na_2_SeO_3_ and BaCl_2_. The mixture is heated at 75 °C for 8 h, neutralized with NaOH, and ethanol-precipitated to obtain selenized polysaccharides (Figure 5) [43].

### 2.5. Methylation

For methylation, polysaccharide powder was completely dissolved in dimethyl sulfoxide (DMSO). Then, NaOH powder was added, supported with ultrasound until complete dissolution. After reacting for 3 h under stirring, methyl iodide was slowly added to an ice-water bath, and the reaction was continued at 4 °C for 3 h. Then, distilled water was added to terminate the reaction. Finally, methylated polysaccharides were obtained using chloroform extraction. Methylated polysaccharides can be detected using FT-IR spectroscopy, and the complete disappearance of the hydroxyl absorption peak at 3200–3700 cm^−1^ proves that the methylation is complete [44].

**Figure 6:**
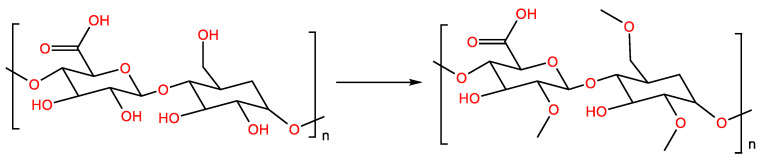
Methylation-modified polysaccharide reaction formula.

The low solubility of polysaccharides in DMSO limits the process of methylation. Polysaccharide dissolution in dimethyl sulfoxide can be supported by ultrasound and microwave radiation [45]. In addition, the heating of polysaccharides in DMSO can increase their swelling and improve their degree of methylation. Polysaccharides’ incorporation in glycerol can also increase the solubility of methylated polysaccharides [46]. In the structural analysis of polysaccharides, the methylation of polysaccharides provides a possibility for the analysis of the connection mode of sugar chains (Figure 6) [47].

### 2.6. Acetylation

**Figure 7:**
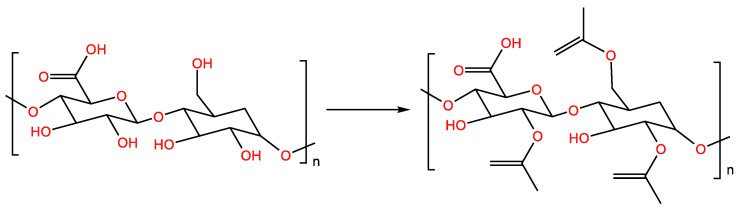
Acetylation-modified polysaccharide reaction formula.

Acetylation modification can change the water solubility and hydrophobicity of polysaccharides [48]. The hydroxyl group of the polysaccharide is converted into an acetyl group through esterification during the acetylation process, and the acetyl group can cause the polysaccharide branch to stretch and change the spatial arrangement of the polysaccharide chain, so that the hydroxyl group of the polysaccharide is located outside the polysaccharide chain [49]. One dissolves the dry polysaccharide powder and acetic anhydride in formamide and mixes the solution well. Pyridine is added dropwise to the mixture and stirred at room temperature for 12 h. Acetylated polysaccharides are obtained via ethanol precipitation and lyophilization using dialysis [50]. In the acetylation process, the choice of catalyst has a great influence on the reaction. Pyridine and 4-dimethylaminopyridine are commonly used as catalysts for acetylation, but their use has decreased due to their toxicity and price. N-bromosuccinimide can catalyze the acetylation of polysaccharide acetic anhydride under mild conditions (Figure 7) [51].

## 3. Structural Characterization of Structurally Modified Polysaccharides

The structural modification of polysaccharides usually involves adding groups to the polysaccharide chain in order to change its structure [52]. However, during the course of the reaction, some reactions lead to significant breakage of the polysaccharide chains and greater changes in the structure of the polysaccharide. One characterizes the structure of the polysaccharide modified using chemical and physical methods to ensure that the expected polysaccharide can be obtained.

### 3.1. Determination of Characteristic Signals

**Figure 8:**
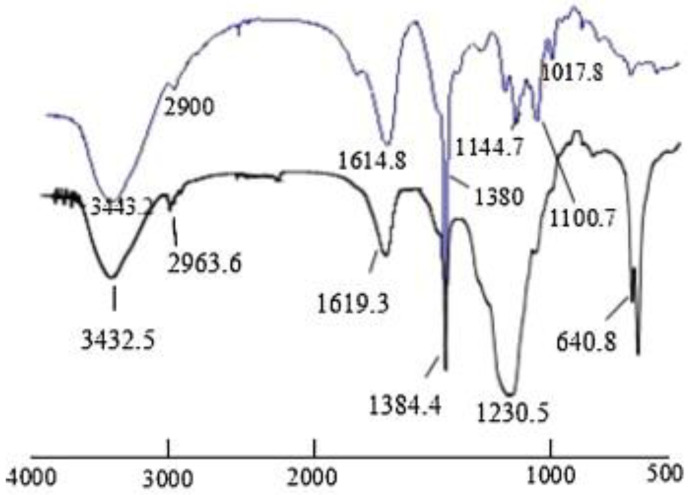
Infrared spectra of sulfated polysaccharides [46].

The modified products of polysaccharides need to be tested via FT-IR to prove the success of the substitution (Figure 8). Table 1 shows the basic characteristics of FT-IR spectra following successful structural modification.

### 3.2. Structural Characterization

The chemical modification of polysaccharides must be performed using purified polysaccharides [55]. High-performance gel permeation chromatography was used to analyze the molecular weight of polysaccharides [56]. The structural information that can be determined in this analysis includes the monosaccharide composition, the position of glycosidic bonds, chirality, the sequence of the monosaccharide residues and their surface features [57].

#### 3.2.1. Ultraviolet Spectrum

Ultraviolet spectroscopy accurately determines the molecular structures of organic compounds. A UV spectrophotometer is used to detect the polysaccharide solution in the range of 200–800 nm, and UV absorption at 260 nm and 280 nm is monitored to determine the contents of protein and nucleic acid [58].

#### 3.2.2. High-Performance Liquid Chromatography

The monosaccharides of polysaccharides comprise an important part of polysaccharide chain composition analysis. Generally, polysaccharides are hydrolyzed via acid solution to break the polysaccharide chains. Each monosaccharide in the polysaccharide is dissociated, and each monosaccharide in the polysaccharide is detected. Monosaccharides undergo no absorption under ultraviolet light, and they are derivatized via PMP and detected using high-performance liquid chromatography [59].

#### 3.2.3. High-Performance Gel Permeation Chromatography

There are certain differences in the biological activities of polysaccharides with different molecular weights. Hydrogels based on low-molecular-weight laminarin have greater application potential in wound dressings [60]. High-performance gel permeation chromatography can be used to analyze not only the molecular weight of polysaccharides but also the homogeneity of polysaccharides [61]. When the homogeneous polysaccharide is detected, it has a symmetrical elution curve, which provides a stable premise for later analysis using gas chromatography–mass spectrometry and nuclear magnetic resonance imaging.

#### 3.2.4. Gas Chromatography–Mass Spectrometry

The connection mode of monosaccharides in the polysaccharide chain is the basis for analyzing the structure–activity relationship of polysaccharides, which can provide a clearer understanding of the structure of polysaccharides [62]. The polysaccharide is modified via methylation, and all the hydroxyl groups in the polysaccharide chain are replaced. Polysaccharide chains are hydrolyzed into individual fragments using volatile acid solutions. Gas chromatography–mass spectrometry is used to analyze the hydrolyzed polysaccharide fragments and resolve the linking sites of each sugar chain. When polysaccharides are acid-hydrolyzed, they can be divided in two ways: complete acid hydrolysis and partial acid hydrolysis. More sugar chains can be obtained using complete acid hydrolysis, and more connection information can also be obtained. However, the location of the complete acid hydrolytic cleavage is uncertain, which may lead to hydrolysis of the structure of the monosaccharide, causing errors in the structural analysis. Partial acid hydrolysis is a milder mode of cleavage, involving the breakage of only the attachment sites of the unstable points in the polysaccharide chain. Although there are fewer ways to obtain monosaccharide chain connections, there will be fewer errors.

#### 3.2.5. NMR

**Figure 9:**
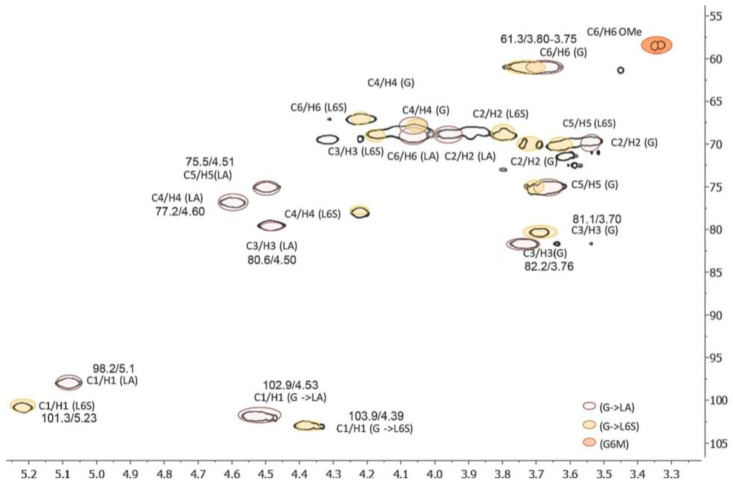
Analysis of sulfated polysaccharide structure via HSQC spectroscopy [64].

NMR can clearly reveal the structure of the modified polysaccharide [63]. Based on a combination of monosaccharide composition analysis and methylation analysis, the chemical shifts generated by anomeric carbon and anomeric hydrogen were analyzed. According to the monosaccharide composition, the one-dimensional nuclear magnetic resonance spectrum was used to analyze the relevant chemical shift, combined with the two-dimensional nuclear magnetic resonance spectrum to analyze the relationship between ^13^C-^1^H and ^1^H-^1^H. Finally, the linking sites between the monosaccharides were determined to obtain the accurate structure of the modified polysaccharides. By comparing the structural changes in the polysaccharides before and after modification, the reasons for the changes in their biological activities were analyzed, which laid the foundation for the study of the chemical modification of the polysaccharides (Figure 9).

#### 3.2.6. Scanning Electron Microscopy

In order to observe the surface structure of a modified polysaccharide more clearly, it can be observed using a scanning electron microscope. Different surface morphologies will have different degrees of influence on polysaccharide physical properties.

Sulfation and phosphorylation may cause the hydrolysis and cross—linking of glycosidic bonds due to their acidic environment, resulting in changes in the connection of glycosidic bonds [53]. ^13^C NMR is widely used in the structural analysis of polysaccharides. The carbon directly connected to the electron-withdrawing sulfuric acid group or phosphate group will move to the downfield region, making the ^13^C NMR of the modified polysaccharide more complicated [65]. At the same time, the carbons indirectly attached to the group will be shifted to higher fields. Dissolving the modified polysaccharide in water can increase the electrostatic repulsion, resulting in a change in the conformation of the modified polysaccharide. The introduction of modifying groups enhances the rigidity of sugar chains and leads to the formation of relatively extended, flexible sugar chains [22].

**Figure 10:**
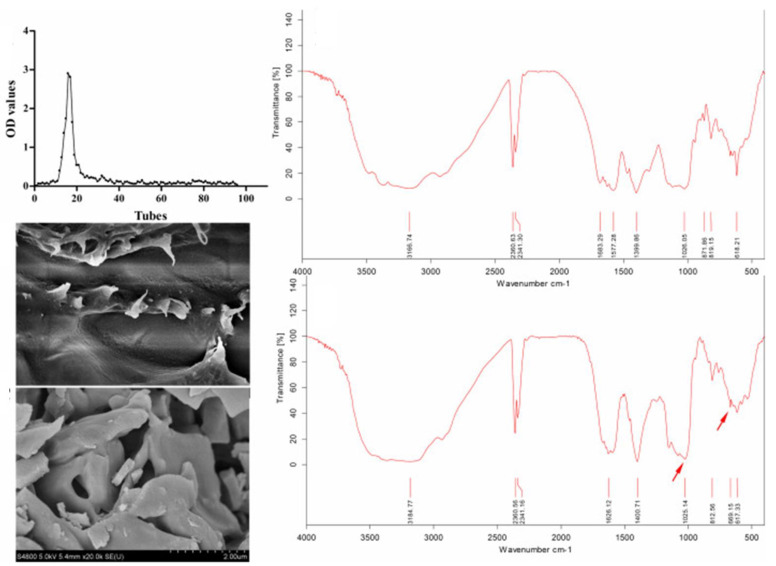
Structural characterization of selenized polysaccharides [66].

Therefore, the structural modification of polysaccharides connects sulfate groups, phosphate groups, carboxymethyl groups, inorganic selenium, methyl groups and acetyl groups in polysaccharide chains, which can not only change the structure of polysaccharide chains but also affect the tertiary structure of polysaccharides, thus affecting the biological activity of polysaccharides (Figure 10).

## 4. Biological Activity of Structurally Modified Polysaccharides

The structural modification of polysaccharides can not only enhance the biological activity of polysaccharides but also add new activities.

### 4.1. In Vitro Antioxidant Activity

#### 4.1.1. DPPH Free Radical Determination

DPPH is a stable free radical, and its scavenging activity is a rapid method for determining the ability of antioxidants to scavenge free radicals [67]. DPPH radical scavenging ability can be significantly increased by introducing sulfate groups or phosphoric acid groups into polysaccharide chains [1]. Sulfated polysaccharides from *Sphallerocarpus gracilis* activate anomeric hydrogen to enhance antioxidant activity [17]. *Pholiota nameko* phosphorylated polysaccharide has a high scavenging ability for DPPH, which is significantly better than that of natural polysaccharides [68].

#### 4.1.2. Determination of Hydroxyl Radicals

Hydroxyl free radicals can easily pass through the cell membrane and react with many biological macromolecules such as DNA, unsaturated fatty acids and proteins in the cell, causing severe oxidative stress damage [69]. Reducing the damage caused by hydroxyl free radicals is achieved through two antioxidant mechanisms: one is to inhibit the production of hydroxyl free radicals, and the other is to scavenge the produced hydroxyl free radicals [70]. Antioxidants may combine with metal ions and react with H_2_O_2_ to form metal complexes. The formed metal complex cannot further react with H_2_O_2_ to generate hydroxyl radicals [71]. Sulfated or phosphorylated polysaccharides can provide hydrogen to combine with hydroxyl radicals to form stable free radicals, thereby achieving the purpose of the scavenging of hydroxyl radicals. The -OH groups of phosphorylated garlic polysaccharides can be replaced with phosphoric acid groups, which have a greater scavenging ability for hydroxyl radicals [72].

#### 4.1.3. Determination of Superoxide Anion

**Figure 11:**
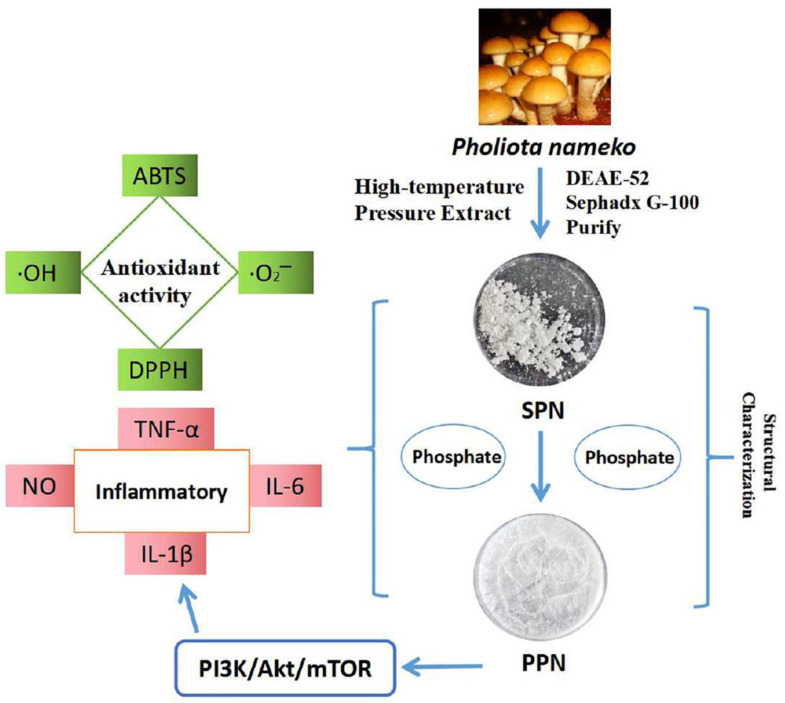
Structural characterization and antioxidative activity of phosphorylated polysaccharides [68].

Superoxide anion is a highly toxic substance that induces hydroxyl radical formation and lipid peroxidation, which can be toxic to biomolecules [70]. Sulfated polysaccharides of Cyclocarya paliurus with higher DS showed a stronger superoxide anion scavenging effect. With the sulfation modification of black currant polysaccharides using the sulfamic acid/4-dimethylaminopyridine method, the sulfated polysaccharides have a good superoxide anion scavenging ability [73]. The scavenging effect of pumpkin polysaccharides and their phosphorylated pumpkin polysaccharides on superoxide anions was determined through pyrogallol autoxidation, and it was found that the phosphorylated pumpkin polysaccharides had a higher superoxide anion capacity [74]. Sulfate and phosphate are both strong electron-withdrawing groups, which endow superoxide anions with higher a hydrogen-donating capacity after modification and improve the superoxide anion-scavenging activity of modified polysaccharides (Figure 11).

### 4.2. Antitumor Activity

Polysaccharides and polysaccharide derivatives can exhibit antitumor activity by inducing apoptosis and arresting the cell cycle [75]. The negatively charged phosphate groups of phosphorylated polysaccharides can bind to receptors on the surfaces of immune cells and activate immune responses, resulting in antitumor activity [76]. At the same time, long-chain polysaccharides increase the possibility of phosphate groups’ binding to the receptors on cells. Sulfated Artemisia globosa polysaccharide can significantly inhibit the growth of HepG2 and Hela cells. Then, the sulfated Artemisia globosa polysaccharides can arrest H22 cells in the S phase and promote cell apoptosis [77]. Selenated mugwort polysaccharide can inhibit the growth of human non-small-cell lung cancer cell lines, and its inhibitory effect is not related to apoptosis or cell cycle arrest, which may be due to its selenium content, promoting antitumor activity in vitro [78]. Ginkgo biloba acetylated polysaccharide has an inhibitory rate of 78.97% for cancer cells [79].

### 4.3. Immunomodulatory Activity

**Figure 12:**
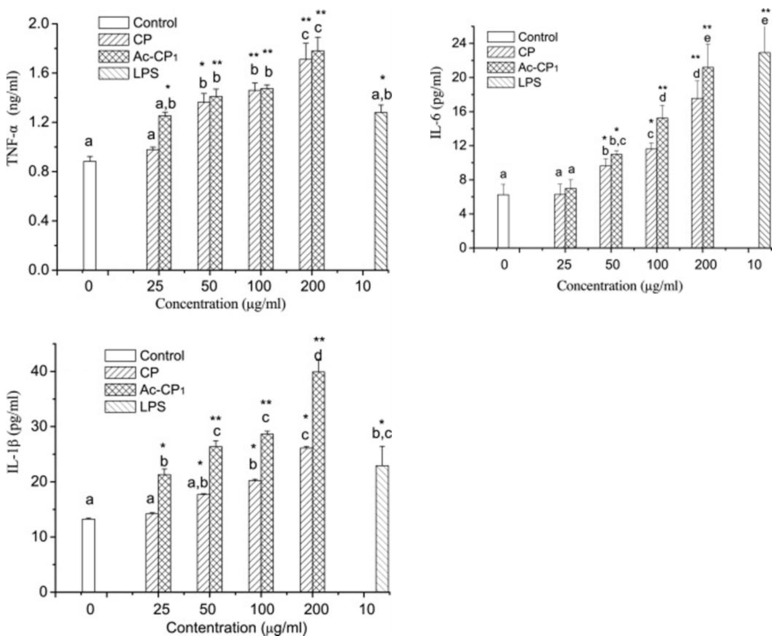
Acetylated polysaccharides of *C. paliurus* can promote IL-1β, IL-6 and TNF-α secretion by mouse macrophages [12].*: *p* < 0.05; **: *p* < 0.01; a, b, c, d, e represents different values, a < b <c < d < e.

The immune system plays the role of traditional Chinese medicine in resisting the invasion of pathogens and maintaining human health. Polysaccharides are immunomodulatory in two main ways. On the one hand, they directly kill pathogens, and on the other hand, they strengthen the immune system by enhancing the activity of macrophages and T lymphocytes [80]. Sulfated polysaccharides can improve the phagocytosis of macrophages and promote the secretion of nitric oxide, reactive oxygen species and various cytokines [10]. Selenization can significantly enhance the immunomodulatory activity of lily polysaccharides, and the strength of this activity is regulated by the ratio between the selenium content and the lily polysaccharides [66]. The acetylated polysaccharides of *C. paliurus* can promote IL-1β, IL-6 and TNF-α secretion by mouse macrophages and, at the same time, promote the synthesis of NO and NO synthase in mouse macrophages and enhance their immunomodulatory activity (Figure 12) [12].

### 4.4. Antiviral Activity

Polysaccharides have antiviral activity, and natural polysaccharides can significantly enhance antiviral activity after modification [29]. Polysaccharides can play an antiviral role by directly killing viruses, inhibiting viral adsorption, inhibiting viral invasion, inhibiting viral deposition and activating the immune system to resist viral infection. Phosphorylated Achyranthes polysaccharides can form a complex with the virion, which occupies the viral envelope site required for viral infection and prevents the virus from attaching to the host cell surface [81]. Selenium deficiency can lead to a decline in human body function and increase people’s risk of contracting highly pathogenic viral diseases [23]. Selenium polysaccharide can inhibit viral infection by preventing the adsorption of the virus. Selenized wakame polysaccharides have good anti-CVB3 activity in the range of 0.5–0.015 μg/mL [82]. Selenium polysaccharides in Eucheuma have a good ability to block viral adsorption so as to achieve an antiviral effect [83].

### 4.5. Antibacterial Activity

Polysaccharides have anti-biofilm properties, which can block the input of nutrients and inhibit the growth of bacteria. The antibacterial activity of sulfated polysaccharides may be related to the antibacterial effect of the sulfo group [84]. Carboxymethylated polysaccharides are more potent inhibitors of Gram-positive than Gram-negative bacteria [85]. Carboxymethylated polysaccharides from Macroalga celiacs also have stronger antibacterial activity. *Dendrobium hancockii Rolfe* polysaccharides modified via acetylation can inhibit the activity of Bacillus cereus, Bacillus subtilis and pullorum [86]. Polysaccharides form complexes with rare earth elements through the chelation of carboxyl groups, which are then added to the polysaccharide chain through carboxymethylation [87]. This inhibits the fungal activity of phytopathogenic fungi.

### 4.6. Anticoagulant Activity

Coagulation is an important method of physiological hemostasis. In recent years, cardiovascular diseases, including heart disease and stroke related to thrombosis, have become the leading cause of death worldwide [88]. For patients with cerebral thrombosis, pulmonary thrombosis and stroke, anticoagulants can prevent the formation of blood clots and maintain health [11]. Coagulation assays based on thromboplastin time, prothrombin time and thrombin time are used to assess anticoagulant activity. Some sulfated polysaccharides have anticoagulant and antithrombotic effects and are nontoxic to normal cells. Heparin is a kind of sulfated polysaccharide, which is widely used clinically as an anticoagulant and antithrombotic agent [89]. Sulfated polysaccharides exhibit anticoagulant activity mainly through the strong negative charge of their sulfate groups and the positively charged amino acid residues on their coagulation factors through ionic interactions [90]. In general, the anticoagulant properties of sulfated polysaccharides are related to the percentage of sulfate, while the anticoagulant properties of phosphorylated polysaccharides are related to the increase in molecular weight, inversely proportional to the percentage of phosphate [91]. Phosphorylated tan ink polysaccharides prolong the partial thromboplastin time, prothrombin time and thrombin time required to exert an anticoagulant effect [92].

### 4.7. Others

Phosphorylated Trichosanthes polysaccharides have significant anti-aging activity in galactose-induced aging mice, and this anti-aging activity may be related to antioxidant and immunomodulatory abilities [34]. *Morchella angusticepes Peck* polysaccharides exhibit a stronger cholesterol-lowering activity after carboxymethylation [93]. *Lachnum* sp. polysaccharides can reduce serum and liver lipids and atherosclerosis and enhance the activity of antioxidant enzymes in mice with hyperlipidemia after selenization [38].

## 5. Conclusions and Outlook

Previous studies have focused on chemical modifications of polysaccharides that can improve their biological activity. In addition, our research found that a modified polysaccharide is affected by its structure and physicochemical properties, such as its water solubility, molecular weight, degree of substitution, main chain and degree of branching. Reagents for the structural modification of polysaccharides are generally toxic, or the steps are cumbersome. After the application of modified polysaccharides, the toxic reagents need to be removed, and the modification method needs to be improved. The structural analysis of polysaccharides is a difficult problem for the analysis of the biological activities of polysaccharides; hence, the structure–activity relationship of polysaccharides and modified polysaccharides will be the focuses of future research. Multidisciplinary or interdisciplinary research is an important trend in the development of modern medicine, involving the combination of modified polysaccharides and medical materials. Not only can such materials act in vivo, but they also provide more possibilities for in vitro applications.

## Figures and Tables

**Table 1:** The infrared characteristic absorption peaks of modified polysaccharides.

Modification Method	The Characteristic Absorption Peak	Reference
Sulfation	New peaks at 1223 and 640 cm^−1^	[10]
Phosphorylation	The regions of 1252–1259 cm^−1^ and 897–911 cm^−1^	[53]
Carboxymethylation	A new signal at 1424 cm^−1^	[36]
Selenization	New peaks at 926 and 840 cm^−1^	[41]
Methylation	A band around 1090 cm^−1^	[49]
Acetylation	New peaks at 2970 and 1379 cm^−1^	[54]

## Data Availability

Publicly available datasets were analyzed in this study. The data can be found here: https://pubmed.ncbi.nlm.nih.gov/ (accessed on 11 July 2023).

## Sample Availability

Not available.
